# Using Social Networking Sites in Research: An Emerging Approach to Engaging With Young People Who Have a Parent With a Mental Illness and/or Substance Abuse Disorder

**DOI:** 10.3389/fpsyt.2019.00281

**Published:** 2019-05-01

**Authors:** Christine Grové

**Affiliations:** Faculty of Education, Monash University, Melbourne, VIC, Australia

**Keywords:** young people with parents with mental illness, social networking sites, mental health practices, recruitment, research with young people, online research methods, youth at risk, research ethics

## Abstract

The challenges involved in engaging young people who have a parent with a mental illness in research and in programs are well documented. Social networking sites provide a potentially useful medium to include at-risk youths and their families by removing some accessibility barriers that may prevent engagement and connection with individuals whose parents or family members have a mental illness. This paper examines how social networking sites can be used to recruit youths and/or their families and engage them in research. Applying a case study analysis, the implications of using social media as a tool for recruitment and data collection and the ethical considerations and limitations will be discussed. Results tentatively indicate that social networking sites may be an effective method to engage young people of parents with a mental illness. The study argues for more informed use of social platforms for the translation and dissemination of research and intervention prevention. Overall, this paper will contribute to public mental health practice through guidelines and policy about social media research with at-risk young people and their families.

## Introduction

Young people who have a parent with a mental illness can experience difficulties throughout their lives, sometimes beginning in their early years. These difficulties can impact on the young person’s well-being and can lead to their own mental health challenges (1). An epidemiological study suggests that around 21–23% of young people have been found to have, or have had, a parent with a mental illness (2). Research consistently reports higher rates of emotional, behavioral, and developmental difficulties in these youths compared with those in the general community (3), with long-term effects including social and occupational issues (1). They may experience increased rates of psychiatric disorders through an interplay of both genetic and environmental factors (4). Self-harm and suicide rates tend to be higher in these youths (5, 6). The young person may harbor resentment towards the parent (7) as well as experience negative emotions including shame, depressed mood, fear of conflicts, loneliness, feelings of abandonment, anger, or envy of peers (8). It could then be said that young people who have parents with mental illness and/or substance abuse disorders are an at-risk group within society. Despite the risks that the young person with a parent with a mental illness may experience, many demonstrate resilience. Researchers agree that some children with a parent with a mental illness can cope well (9), acquire and practice effective problem-solving skills (10), and access functional social supports external to the family (9). Young people need protective factors, for example, adequate community support and intervention (11) and accurate mental health information (12), to reduce the impact of negative risk factors. In this paper, young people or youths are considered to be aged between 14 and 18 years old.

Children of parents with a mental illness are aware that there are some predictable and unpredictable elements of their parents’ behavior, and it is the unpredictable elements that often impact the young person the most (13). Their understanding of parental mental illness changes with age as well as with the amount and type of information about mental health they acquire (14). Slominski (15) measured adult children’s perceptions of growing up in a family where a parent was diagnosed with a mental illness. He, too, proposed that it was not only the experiences themselves that shaped the individual but also the way the individual made sense of those experiences. When young people do not receive complete, accurate mental health information, they may come to their own—often inaccurate—conclusions that can exacerbate their fears. A general lack of awareness of their parent’s condition may negatively influence the youth’s ability to access systems of support. Some young people make attempts to obtain information from the mental health professionals who care for their parents, but many report that they do not get the recognition they feel they deserve, for example, a lack of acknowledgment of the young person’s role in caregiving and their need to understand what is happening for their parents and themselves (7, 16).

While extensive research has identified young people’s risk of adverse outcomes and the potential for resilience (2, 17), it is challenging to recruit young people and families where a parent has a mental illness in research and into prevention programs (18). The difficulties of engaging with youths from high-risk communities in research may be due to barriers in recruiting vulnerable youths (19, 20). These barriers can include i) insufficient understanding of research (21, 22), ii) mistrust of research (21), iii) stigma associated with participation in clinical research (23), and iv) issues surrounding parental consent (23). There are few papers that outline “adolescent friendly” recruitment strategies that can be employed to overcome barriers to recruitment and effectively engage with at-risk young people in research initiatives (24). A potential way to address these barriers can be to recruit at-risk youths through the mediums in which they connect with most—that is on the Internet *via* social networking sites (25). How social networking sites are used to recruit youths and/or their families and engage them in research should be explored, as well as the potential to use social networking sites for translation and dissemination of accurate public health information. This paper is the first attempt to examine social networking platforms as a potentially useful medium to include at-risk youths and their families by attempting to remove barriers that could prevent access to and involvement of individuals in research.

## A Technology-Based Response: Social Networking Platforms as a Potentially Useful Medium to Include At-Risk Youths and Families

Social networking platforms are online communication systems that facilitate community-based contributions, interactions, content sharing, and collaboration in virtual networks *via* social media websites Facebook, Twitter, blogs (i.e., Tumblr), and/or YouTube (26). Social networking sites have potential when recruiting a younger population as well as targeted groups of individuals (24), such as youth with a parent who has a mental illness. For some young people, the Internet is their primary source of information and their main means of communication with the world (25). There has been a large growth of online social connections: Facebook has around 1.59 billion monthly users (Facebook 2018), while on Twitter around 500 million tweets are sent per day (Twitter 2018). Social media is increasingly an information source for youths, where they use websites to learn about the world and themselves and connect with others (27). Young people are more inclined to use social networking sites than older adults (28). As a result of the rise in social media usage, researchers and policymakers have begun to consider how such platforms can be harnessed to support research, share accurate public health messages, and inform robust evidence-based policymaking.

A systematic review of 25 studies that employed Facebook to recruit young people (10–18 years old) found that paying Facebook for advertising about specific research projects was a successful tool for recruiting adolescent participants into mental health research (24). However, the review noted that there have been few studies in which underrepresented or at-risk adolescents have been recruited *via* social media platforms (24). There have been no studies that examine social media use in recruiting children and families where a parent has a mental illness. There is relatively little guidance on using online platforms in social and mental health research. Further research is clearly needed particularly given that social networking sites are considered to be an emerging data collection, recruitment, and research dissemination strategy that is gaining traction in research (25, 26, 29).

Social networking sites can be a useful tool for gathering data, which has the potential to address barriers in research. For example, they can be a platform to deliver health interventions and recruit participants for studies in a cost-effective manner (25, 26, 29). Some of the barriers to recruiting and engaging young people who have a parent with a mental illness in research may be addressed through social platforms. Ongoing discussion about the purpose of social media methods is necessary to identify how social networking sites are applied across individual research studies. This will contribute to the field’s understanding of the ways in which these platforms can be used to access at-risk groups in the community (19). There are, however, several factors to consider, such as whether to use social media research as the sole method or in conjunction with other methods, processes of data collection and analysis, ethical implications of consent and privacy, and the presentation of social media research findings. There is a lack of policy and guidelines for using social networking sites in research (19). This paper will apply a case study analysis of a research project that used social media as a strategy to recruit young people who have parents with a mental illness and to translate and disseminate research knowledge.

## Method and Results of Case Study

Stage 1 of the research project was to explore whether a social networking site could be used to recruit and engage with youths whose parents have a mental illness (the focus of this paper). Stage 2 gathered youth people’s experiences of help seeking using a mixed methods approach (16). The objective of stage 2 was to identify their expressed preferences for supports (for example, informal supports such as talking to peers or parents) and more formal interventions (such as developed programs). In order to answer this research focus, a self-administered questionnaire followed by individual semi-structured interviews was employed. In total, 173 young people (13–17 years) who have a parent with a mental illness completed a questionnaire and 6 of these participants took part in interviews. Initially, 175 youths were recruited; however, 2 in the sample were male and subsequently removed, leaving the sample of females only. During stage 1 of the study, the social networking site Facebook was used in two ways: i) as a recruitment strategy in response to the research objectives and questions, and ii) as a technology-based mechanism for knowledge translation and dissemination with the aim to provide accurate and helpful information about mental health and support for young people and their families.

### Online Research Presence

**Figure 1:**
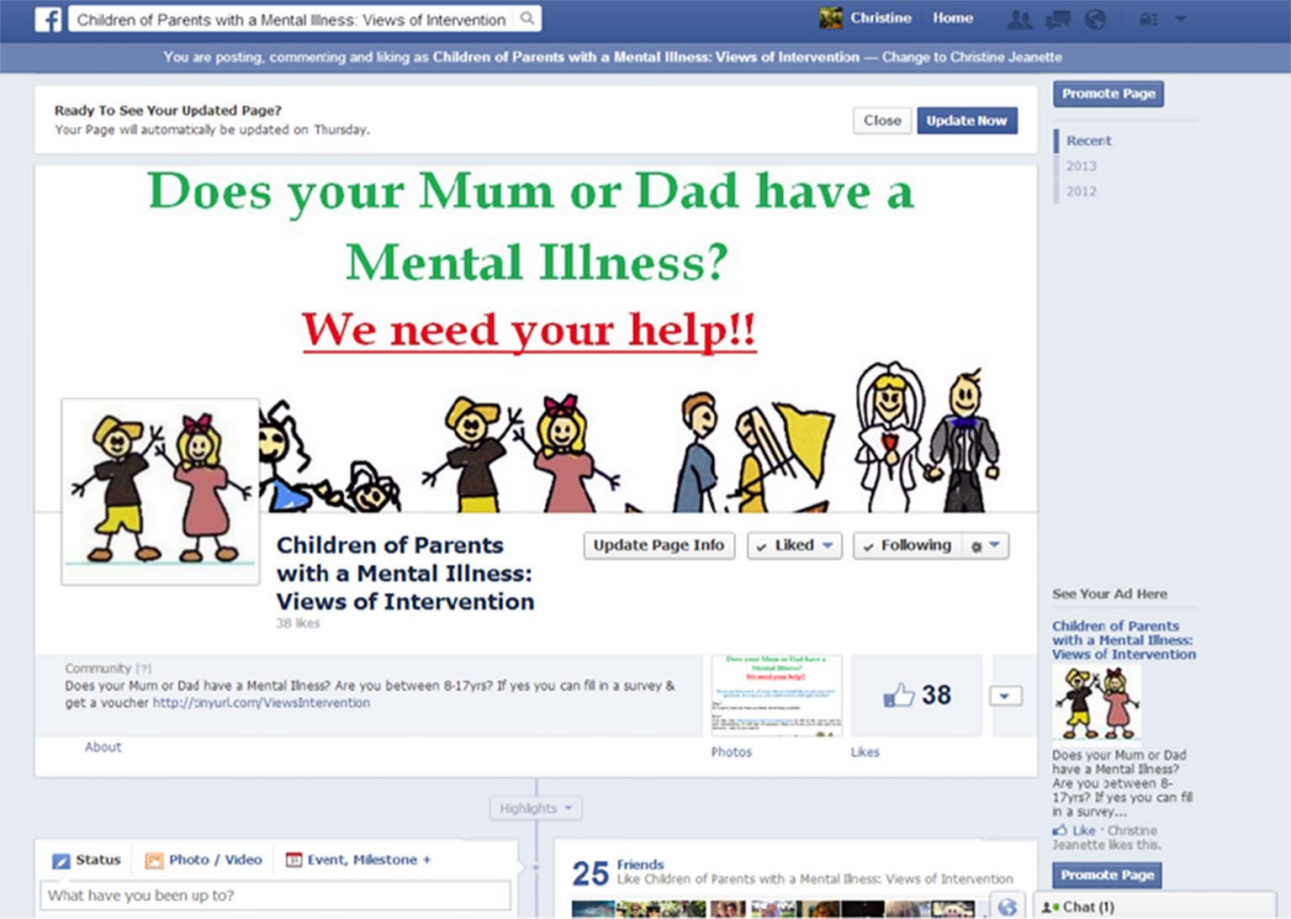
Facebook research page.

A Facebook page was created to provide participants with information about the research project (see **Figure 1**). It was titled “Children of Parents with a Mental Illness: Views of Intervention.” The main objective of the Facebook research page was to disseminate the online questionnaire, promote awareness of the study, and disseminate public mental health information. Posts made on the Facebook page included information about the research team and project, support services, the latest research about mental health and well-being, and study announcements that included a link to the study questionnaire. On average, the lead researcher made two Facebook posts a week. During the recruitment period, the researcher made 100 Facebook posts, received 75 Facebook followers, and liked 78 organizations relating to young people and mental health. The lead researcher reviewed and approved all wall posts in a timely manner and took them down if inappropriate due to swearing or cursing. The author interacted with other Facebook pages by “liking” the pages of community organizations. The organizations were found using keyword search terms on the Facebook search engine, such as “children of parents with a mental illness,” “young carers,” “youth mental health,” and “youth well-being.” The “About” section on the Facebook research page included detailed information about the study, the researchers’ names and affiliations, and links to the researchers’ official affiliation website.

### Advertising Research on Facebook

**Figure 2:**
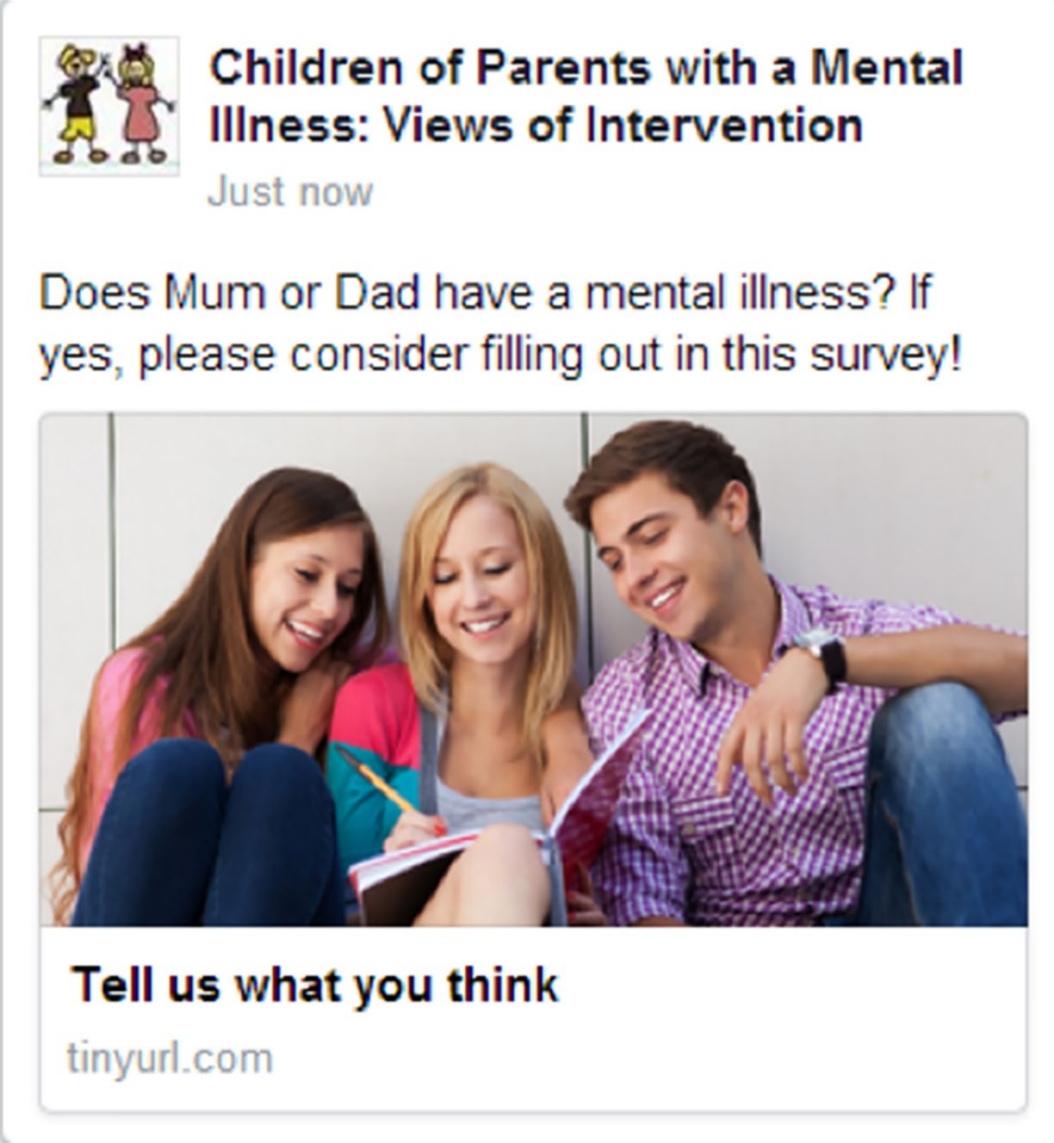
Example of advertisement used.

The researchers paid for targeted advertising to meet specific criteria. The criteria included the age group of the targeted youths (13–18 years), a specific population (children who have parents with a mental illness), and geographical location (Australia only). The study advertisements were only presented to Facebook users who met the study criteria set by the researchers *via* the Facebook administration page. It was shown to potential participants on their personal Facebook pages *via* their desktop computer and/or mobile device. Advertisements on Facebook must adhere to language and image guidelines. The headline had a limit of 25 characters, and the body text of the advertisement had a limit of 90 characters. Images were required for all advertisements. The study advertisement (see **Figure 2**) was placed on Facebook from August 2013 to February 2014, inviting youths to participate. Those who clicked on the poster were directed to the research explanatory statement at the beginning of the survey on a survey website called Qualtrics. Consent was obtained from the young person when they agreed to complete the questionnaire through an online form at the beginning of the questionnaire. Participants were requested to obtain parental consent before completing the survey; this request was on each page in the survey (e.g., “Ask your parent if you can take part in this study”).

A total of 1,382 people clicked on the link to the questionnaire on the advertisement on Facebook. Of those who accessed the website link, 192 provided consent. Of these, 175 met the study inclusion criteria and subsequently completed the questionnaire. No other methods of data collection (such as mail-out surveys) were used. Of the 175 samples, 6 participants agreed to take part in an individual semi-structured telephone interview and were offered a $10 gift voucher upon completion. The study found that the use of social media, specifically Facebook, was a more effective recruitment tool than using traditional methods (such as flyers or mail-out surveys) to recruit young people of parents with a mental illness (12, 16).

### Online Snowball Sampling

Snowball sampling occurred online during the recruitment phase of this study. Snowballing transpired when community organizations shared the Facebook research page and the survey on their websites and social media pages. From there, a snowballing effect occurred: the Facebook research page and posts made by the author were distributed *via* social media through community organization followers (individuals who followed the page of the organization). These were then subsequently shared by individuals on their individual social network site, that is, on blogs, in personal newsletters, and/or on their Twitter/Facebook pages. In addition, online snowball sampling occurred through group and friendship connections between Facebook users, where the study details were shared further.

## Discussion

There are benefits and challenges of using social media as a recruitment tool when engaging with vulnerable youths in research. Some benefits include using a platform that is widely interacted with by young people, whereby the research can be shared across many social sites within a young person’s network, enabling a questionnaire to be disseminated quickly among many potential participants (30, 31). Amon and colleagues (24) suggest that advertising on social networking sites alongside traditional means of recruitment (i.e., flyers, mail-outs, and telephone calling) could be helpful in accessing larger and more specific groups. Social media presents a fast, affordable method of recruiting study participants for questionnaire research. In the current study, using social media was found to be a successful strategy to recruit a large sample of young people who have parents with a mental illness in a short period of time (16). Clearly, online recruitment for structured questionnaires *via* social media is a feasible and efficient tool that can be used to access at-risk individuals.

The Australian Psychology Society (32) released a national survey examining the impact of technology and social media on the well-being of young people (aged 14–17 years old). The survey found that young people are self-reporting that they use social media for an average of 3.3 h each day, on five or more days of the week. The vast majority of teenagers reported that their screens and social media accounts were a positive part of their lives. Many use social media channels to connect with family and friends and to entertain themselves. Despite social media playing a positive role for most, the high use of social media and technology can have a negative impact on youth self-esteem. Two in three young people feel pressure to look good, and nearly a third of youths have been bullied online (32). Clearly, there are ethical and potential limitations when using social networking platforms in practice or research that should to be taken into account.

Young people and their families from rural or lower socioeconomic status have been found to be more difficult to recruit and engage than those from higher socioeconomic status or from urban areas (33). This may be due, in part, to disadvantages associated with living in these areas that include reduced quality of services (34), but can also be attributed to the challenges that coincide with living in poverty or far from central towns including lack of a reliable telephone service and limited or nonexistent access to the Internet (33). Thus, a possible limitation is that participants recruited through social media may not be representative of the population as not all young people have access to computers or the Internet. Some research suggests that the digital divide is reducing as more individuals are online; however, age is a crucial factor. Nine in 10 people aged between 15 and 54 use the Internet; however, this statistic reduces to 8 in 10 of those aged 55–64 years, and to under 6 in 10 of those over 65 years (35). The age of young people who can be recruited *via* social platforms is limited to those over the age of 13. Due to age restrictions, individuals 13 years and below cannot create a Facebook, YouTube, or Tumblr account.

A challenge of social networking sites is that the participants are not representative of the population. The findings here thus might not be generalizable to the wider population. It could be that the population is not representative because children of parents with a mental illness may search for information elsewhere than on social media platforms. A predominant limitation of social media for collecting data is that it may introduce self-selection bias. Self-selection bias can lead to a sample bias. Self-selected and self-reported data may affect the reliability and validity of results (36). The validity of the completion of the online survey should be considered, as members of social media sites may not be truthful in their responses and/or complete the survey as a “fake” participant. The online identity of a person may not be the same as their real-life identity. Often, social media identities are to a certain extent more “positive.” This limitation could be helpful, though, if the research is focused on a particular group that is active on a social media platform, and there is the potential to gather data from participants who may not tend to respond to other types of research methods (37). The anonymous nature of online recruitment can be a challenge in that researchers may not be aware if a young person is upset or triggered by the content in the survey. Researchers are not able to check or provide more information if the youth does not understand the project. The Facebook research page seems to have multiple purposes, which makes it difficult to disentangle to what extent this medium is useful for research participation versus general information versus referral or information to other prevention services. For example, the current case study recruited mainly females; thus, the findings or suggestions here may not be appropriate for the recruitment of males. While Facebook is still one of the most used networks in general, currently among some young persons’ Facebook is not their only or main social site they access, when compared to other platforms such as Snapchat, Instagram, or YouTube (38).

Rarely are there papers that include strategies for recruiting at-risk youths (18, 24). Effective recruitment strategies are needed as it is widely recognized that underrepresented adolescents can be challenging to recruit due to barriers that impact their participation. A barrier to youth participation is the way in which at-risk adolescents are recruited. Consent for youths to participate in a study is usually attained from an adult in the young person’s life, such as a parent or guardian, known as a “gatekeeper.” The parent/guardian is not typically the intended audience for a study directed at youths, yet recruitment strategies are often focused at connecting with the “adult gatekeeper” in order to attain consent for the young person’s participation. The challenge in accessing youths *via* a “gatekeeper” is that two sets of individuals are to be recruited for the study and the researchers usually need the parent/guardian to be interested in the research and then the youth. Depending on the study considerations required in recruiting vulnerable youths, such as whether the young person themselves can self-select into a study by providing their own consent, young people can then have “buy in” into a project in that they make the decision to participate in the study. The young person then becomes the “gatekeeper” who chooses to share study information with their parent/guardian, potentially building trust and respect of the young person. This approach respects autonomy and privacy of the young person, who, as noted by the United Nations Rights of the Child under the Convention on the Rights of the Child (39), can decide and choose to take part in matters that directly impact them, and that includes their inclusivity in firstly opting into developmentally appropriate research. A “youth-centered approach” has ethical justification. Some researchers advocate, however, that “adult gatekeepers” are a necessity in that they protect the adolescent from exposure to information that may not be appropriate (40). However, first and foremost, researching alongside at-risk youths requires procedures for the researcher to be aware of, or sensitive about, the “risk status.” An understanding and sensitivity is required of researchers regarding the information they are gathering and to be done so in ways that convey respect for the young person and their circumstance, for example, using a non-judgmental attitude, respect of opinions, and collaborative approaches to consult with young people. A paradigm shift is needed for some whereby youths are seen as experts on their lives, where they have the right to share their perspectives and to initially choose to engage in research.

An ethical consideration when removing “adult gatekeepers” is regarding the ability of minors to provide consent. In the study by Grové et al. (16), formal consent was required from youths only and formal consent was not required from the parents. However, youths were invited throughout the online questionnaire to inform their parents of their participation in the study. The young persons were reminded that they did not have to be involved in the research if they chose not to. The gatekeepers were not in the role of insisting or convincing a student to participate in the research project.

Some youths express concern about identifying or sharing their at-risk status, such as being a child of a parent with a mental illness. If youths are recruited in community or public places, such as at school or through an organization, they know, and are fearful others know, they are being sought for a problem or issue and may not indicate their interest. A fear of exposure of their at-risk status and a lack of privacy are a barrier to youths participating. Social networking sites attend to their concern in that they can take part in the research anonymously and privately. Respect for youth privacy and autonomy, again, are key to recruiting and retention (41) whereby youths view the research as “something interesting and not because of a problem.” However, anonymity can be a challenge in that researchers may not be aware if a young person is upset or potentially triggered by the content in the survey or if the youth has the language to understand the research. Young people who participate in preventive interventions may not necessarily want to participate in research or vice versa. Social media could help with intervention recruitment and delivery—an area that future research could explore.

Research using social network sites is a relatively new phenomenon. Social networking sites appear to be suited to recruiting a younger population who regularly use these platforms and also may be hard to reach, for example, young people of parents with a mental illness and/or substance abuse disorder (18). More generally, the emerging role of social media in research raises ethical concerns:

- privacy of participants (Are their IP addresses identifying)?,
- ownership of the data gathered (Do the data belong to the third-party website as well as the research team? What is done with these data and who are they shared with)?,
- participants providing informed consent (Is informed consent needed when conducting research studies on public websites and social media platforms and, if so, how should the researchers attain this consent)?, and
- access to participants (Who has access to which participant groups in the community and which participants are being excluded)?.

Research that uses social media platforms may consider guidelines for social networking platforms in research that are specific to their study and its context. The guidelines may outline the benefits and limitations of interacting with social media, including the relevant ethical, legal, and governance issues. The practice guidelines will depend on the sensitivity of the research area and if privacy and confidentiality (or lack of) can be provided to participants. For example, depending on the social media user’s privacy settings, posts that they follow, like, or comment on can be seen by others online, creating a digital footprint or trail of interaction. Researchers may also choose to monitor and record the frequency of occurrence of the online snowball sampling strategy to determine its impact.

Researchers may consider whether the method of social media recruitment and dissemination is the appropriate tool to use, with attention to ongoing and regular interactions by the research team of content and public communication within the platform. Given the infancy of social media research, researchers need to make methodological decisions based on previous case study illustrations of research using social media rather than prior personal experience. Social media methods often make use of existing, publicly available data, so the need for participants to complete demographic questions can be reduced. However, existing data may not be accurate and ethical discussions of whether consent should be provided for its use are warranted. Researchers should provide information about their project and the research team publicly, including the research purpose and how the data gathered will be used, stored, and accessed. Ways to protect the interests of vulnerable populations in the community, such as young people who have a parent with a mental illness and/or substance abuse disorder, need to be included in project-specific guidelines. The guidelines may choose to provide suggestions about support, promote help seeking, and disseminate summary information gathered during the study. More research is vital to determine how scholars can use social media to explore participants’ understanding, engagement, and active participation in public mental health practice guidelines and policy.

A challenge with some of the research with children of parents with a mental illness could be that many participants are already connected to support networks. Often, they are recruited *via* this connection. Unless the research aims are to examine outside of this network specifically (recruiting external to support networks, in rural areas, or those not accessing support), it can otherwise be challenging to recruit. Using a social networking site (i.e., Facebook) to aid in recruiting participants outside associated, connected networks did help in addressing this bias. However, if researchers are recruiting vulnerable families who may not be connected to support, educational information about supports (i.e., online or telephone counselling services or how to connect with local doctors) should be provided.

## Conclusion

There are challenges in connecting with and engaging with young people in research, particularly if the young person is considered underrepresented in the community. An untapped approach in attaining youth involvement in research can be through social networking sites. Social networking sites are platforms that young people use directly and regularly, and could be used as a possible recruitment tool depending on the research study objectives. Further research will do well to illustrate the use of social media in research, the risks and benefits, and how efficacious this strategy is in recruiting different at-risk groups of youths.

## Ethics Statement

Ethics for this study was approved through the relevant Monash University committee. Ethical approval number is MUHREC CF13/1766-2013000918. Paid advertising on Facebook was used to recruit participants. Potential participants were shown the information that is on the recruitment poster as an advertisement. The Facebook recruitment advertisement that was shown to potential participants comprised of 1) short title (e.g., “Tell us what you think”), 2) an image (e.g., stock image of young people), and 3) main text, up to 74 characters in length: “Does Mum or Dad have a mental illness? If yes, please consider filling out in this survey!” (See **Figures 1** and **2** for an example of the advertisement used for mobile device and desktop computer). If participants chose to take part in the project, they then clicked the advertisement. This took them directly to the explanatory statement and participant consent forms for participants to take part in the study.

## Author Contributions

The author developed the conception and design of the research work, conducted the data collection, the data analysis and interpretation, drafted the article, provided critical revision of the article, and examined the final approval of the version to be submitted.

## Funding

The author declares that the research was conducted in the absence of any specific funding.

## Conflict of Interest Statement

The author declares that the research was conducted in the absence of any commercial or financial relationships that could be construed as a potential conflict of interest.
